# Measurement and Analysis of 4G/5G Mobile Signal Coverage in a Heavy Industry Environment

**DOI:** 10.3390/s24082538

**Published:** 2024-04-15

**Authors:** Ladislav Polak, Jan Kufa, Roman Sotner, Tomas Fryza

**Affiliations:** Department of Radio Electronics, Faculty of Electrical Engineering and Communication, Brno University of Technology, Technicka 3082/12, 616 00 Brno, Czech Republic; kufa@vut.cz (J.K.); sotner@vut.cz (R.S.); fryza@vut.cz (T.F.)

**Keywords:** 4G, 5G, coverage mapping, industrial factory, key performance indicators, mobile networks

## Abstract

In the evolving landscape of Industry 4.0, the integration of advanced wireless technologies into manufacturing processes holds the promise of unprecedented connectivity and efficiency. In particular, the data transmission in a heavy industry environment needs stable connectivity with mobile operators. This paper deals with the performance study of 4G and 5G mobile signal coverage within a complex factory environment. For this purpose, a cost-effective and portable measurement setup was realized and used to provide long-term measurement campaigns monitoring and recording several key parameter indicators (KPIs) in 4G/5G downlink and upload. To support the reproducibility of the provided study and other research activities, the measured dataset is publicly available for download. Among others findings, the obtained results show how the performance of 4G/5G is influenced by a heavy industry environment and of the time of day on the network load.

## 1. Introduction

In the era of Industry 4.0, the seamless integration of advanced technologies has become essential for the efficient functioning of modern manufacturing facilities. At the heart of this integration lies the reliance on robust and reliable mobile communication networks, particularly the gradual transition from fourth to fifth generation (4G and 5G) technology. As factories embrace the connectivity and speed promises of 5G networks, there arises an imperative need to assess and optimize mobile signal coverage within these dynamic and often complex environments [1,2,3,4,5,6].

“Smart” factories, but also traditional manufacturing facilities and industrial complexes, necessitate a more suitable technical platform capable of handling a high volume of data, such as that obtained from various sensors, while ensuring high reliability. In pursuit of this goal, mobile and/or wireless networks play a crucial role. As these networks gain traction, understanding aspects such as signal coverage, reliability, and capacity becomes paramount for performance optimization and seamless connectivity in demanding environments, like factories with a complex environment (e.g., heavy industry). The measurement and monitoring of 4G/5G mobile signal coverage inside a factory emerge as critical considerations for ensuring seamless communication and connectivity. Often, professional hardware (HW) equipment and software (SW) tools are required to facilitate data collection and analysis. Moreover, datasets from these measurements are not always complete or publicly available, hindering the reproducibility of analyses or their use for optimizing mobile connections in other factory settings [4].

In this paper, we introduce a cost-effective measurement setup that enables us to conduct an extensive analysis of 4G/5G mobile signal coverage in a complex factory environment. Our approach aims to provide comprehensive insights into mobile signal coverage within such a unique transmission environment.

Furthermore, to facilitate the reproducibility of our analysis and support future research endeavors, we have made the complete dataset publicly available. This dataset includes detailed measurements and observations, allowing other researchers to validate our findings and explore additional optimization strategies for mobile connectivity in similar factory environments.

This paper is structured as follows. Section 2 presents an overview of the state of the art and defines the main contributions of this work. Section 3 introduces the created measurement system and provides insights into the indoor environment of the factory where the long-term measurement campaigns were conducted. The analysis and discussion of the obtained results are presented in Section 4. This paper is concluded in Section 5.

## 2. Related Works and Original Contribution

In recent years, numerous works have investigated 4G/5G mobile signal coverage in various indoor and outdoor environments [3,4,5,6,7,8,9,10,11,12,13,14]. However, conducting such studies in harsh or heavy industry environments has received comparatively less attention.

Haq et al. [3] present the findings of a study focusing on customer satisfaction based on 3G/4G network performance in the Punjab region of Pakistan. Their study examines various aspects such as network coverage, customer services, quality of video calls, and download speed. However, details regarding the transmission environments (indoor and outdoor) are not provided, and the obtained dataset is not accessible.

The study presented by El-Saleh et al. [4] provides comprehensive mobile coverage measurements conducted in both indoor and outdoor urban areas of Malaysia. Data, coming from five mobile operators supporting 3G and 4G technologies, were collected through drive tests. The results were evaluated in terms of key performance indicators (KPIs), completed by performance metrics such as throughput, ping, and handover. The findings confirmed the necessity for mobile operators to enhance data rates to facilitate high-speed transmission and low-latency communications. However, it is unfortunate that the dataset for further research analysis is not available.

Rodriguez et al. [5] proposed an experimental framework for integrating 5G wireless systems into industrial applications. The framework was tested in a real small-scale industrial factory environment called the “Aalborg University 5G Smart Production Lab”. During the tests, over 100,000 samples were collected to provide statistical-based performance analysis, focusing on data traffic and packet inter-arrival times; while the study provides valuable insights, it does not give significant attention to investigating the influence of the transmission environment on the monitored system parameters. In [7], researchers conducted a similar experiment using the same industrial environment, this time focusing on the performance of Wireless Fidelity (Wi-Fi) for Industrial Internet-of-Things (IIoT). They utilized a commercial enterprise-grade Wi-Fi 6 system to collect data on parameters such as Received Signal Strength Indicator (RSSI), round-trip time (RTT), and handover. The results highlighted that the location of access points (APs) and different industrial scenarios can have a significant influence on the values of the monitored parameters.

In [8], Rekoputra et al. examined the performance of the 5G network in the Industry 4.0 Implementation Center (NTUST), focusing on parameters such as network speed, latency, jitter, and packet loss rate. To conduct this analysis, advanced measurement equipment was employed, allowing for up to 100 users in a virtual environment. The study emphasized the importance of the technology used in the wireless network, such as Wi-Fi 6 and Ethernet, for enabling a smart factory environment.

Lyczkowski et al. [9] presented a measurement-based performance comparison between a private Long-Term Evolution (LTE) network and an IEEE 802.11b/g/n network deployed in an automotive factory. The measurements focused on monitoring several KPIs while production was ongoing. However, details about the factory environment are not provided, and the dataset is not publicly available. Based on the results, the LTE-based network demonstrates significant advantages over the WLAN-based solution for factory automation use cases.

Schmieder et al. [10] and Mi et al. [11] conducted channel characterization studies in indoor industrial environments, specifically, a high-precision machining workshop hall and a smart warehouse, respectively, utilizing the 28 GHz millimeter wave bands. Their analyses included the examination of path loss and power delay profiles. The findings revealed the unique nature of these industrial environments in terms of wireless communication.

Raida et al. [12,13] reported comprehensive and long-term measurements of a 4G-based LTE network, conducted in Austria, encompassing both indoor and outdoor environments. The studies include a detailed analysis and evaluation of the measured data. Importantly, the dataset resulting from these measurements is publicly available. The authors of both studies have indicated that their dataset can be utilized for research purposes or by mobile operators to optimize the sampling process and measurement duration of KPIs. The analysis of the results revealed the impact of various transmission conditions, such as weather conditions like rain, on the stability of certain KPI measurements.

In [14], Mahmud et al. conducted an analysis of 4G/5G networks, focusing on parameters such as total received data, packet losses, and re-transmissions. The study predominantly relied on simulations and centered on evaluating the performance of different schedulers utilized in 4G/5G networks. One of the key findings of this study is the establishment of a direct relationship between signal-to-interference and ratio (SINR), throughput, and packet losses measured at the user equipment (UE).

### 2.1. Summarizing

From the brief overview presented above, we can conclude the following:

1. Extensive and long-term measurement-based studies of 4G/5G mobile signal coverage in complex (heavy) factory environments have not been reported so far; while studies such as [5,7] have explored similar topics for 5G and Wi-Fi systems, they were conducted in university-based small-scale industrial factory environments.
2. The studies primarily focus on monitoring and analyzing selected KPIs. Additionally, the influence of key environmental circumstances, such as the time of day on network load and on the quality of radio connections in wireless communications, is often not thoroughly investigated.
3. In numerous cases, datasets from measurements are not publicly available, hindering the reproducibility or extension of the research. Complete datasets are only open in works [12,13,15]. However, these datasets are obtained from measurement campaigns in non-factory environments.

### 2.2. Original Contribution

The main contributions of this paper are as follows:

1. We propose a simple and cost-effective hardware (HW) setup that enables long-term and repeatable measurement of KPIs of 4G/5G mobile signals. The measured parameters can be saved and processed offline (a part of them online). Additionally, our concept allows users to remotely connect to the measurement equipment to change measurement configurations or send (save) data. The equipment used is characterized by its small size, portability, and remote connection capability.
2. We conducted an extensive KPI-based performance study of 4G/5G mobile networks, focusing specifically on mobile signal coverage within a heavy industry environment. We explored the potential of 4G/5G mobile networks for data transmission in such a challenging transmission environment. Detailed measurement campaigns were conducted at various locations within the factory, located in the Czech Republic, providing insights into 4G/G5 network performance.
3. To promote the reproducibility of our analysis and support future research endeavors, particularly in the field of artificial intelligence, we made the full dataset publicly available. Our work provides a large public dataset comprising real-world measurements captured at multiple locations within the factory environment. The dataset is openly accessible on GitHub [16].

## 3. Measurement in the Factory Environment

In this section, we introduce our measurement equipment and describe the measurement methodology, as well as the metrics and software (SW) tools used to set up our performance evaluation of 4G/5G mobile signal coverage in a harsh factory environment. The proposed concept is designed to allow repeatable and automated measurements. It can be used for measuring 4G/5G signal quality metrics in various environments, not limited to factory settings, and is independent of the time of day.

### 3.1. 4G and 5G Mobile Networks in the Czech Republic

As mentioned, the measurement campaigns were conducted in a factory located in the Czech Republic. Presently, mobile operators in the Czech Republic support 2G, 4G, and 5G mobile systems. In the case of 5G, operators deploy 5G networks using various radio frequency (RF) bands within Frequency Range 1 (FR1). Specifically, the following RF bands are utilized: 700 MHz (5G), 800 MHz (4G), 1800 MHz (4G and 5G), 2100 MHz (4G and 5G), and 3500 MHz (5G) [17]. These RF bands were selected based on their technical characteristics and coverage optimization. It is crucial to note that all realized 5G radio access networks (RANs) are integrated with existing 4G networks, operating under non-standalone (NSA) architecture. This integration allows for seamless connectivity and enables a smooth transition to 5G technology while leveraging the existing infrastructure of 4G networks [18].

The 700 MHz and 800 MHz RF bands are ideal for wide coverage and penetration into indoor spaces. These RF bands are suitable for achieving extensive area coverage and optimizing signals in challenging environments with physical obstacles. Operators utilize a bandwidth of 10 MHz in this RF band. The 1800 MHz RF band offers mobile operators the ability to provide higher data rates and is optimal for urban areas with higher user device density. In this case, operators utilize a bandwidth of 20 MHz, enabling efficient data transmission in areas with increased demand for broadband connectivity. To establish mobile connections in strategically significant urban areas and locations with high demand for broadband connectivity, operators employ the 2100 MHz and 3500 MHz RF bands. In the 2100  MHz and 3500 MHz RF bands, operators utilize a bandwidth of 20 MHz and 40 MHz, respectively.

### 3.2. Hardware Setup

**Figure 1:**
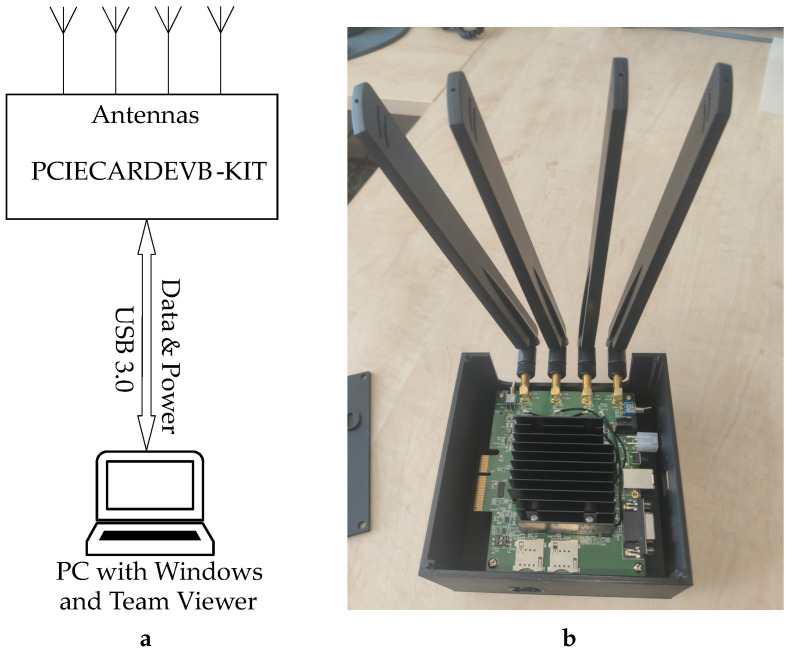
The measurement equipment: (**a**) the block diagram of the proposed measurement system, (**b**) the measurement board used to collect data for analyzing 4G/5G network performance.

The block diagram of the proposed measurement system and a photo of the measurement board used to collect data for analyzing 4G/5G network performance are depicted in Figure 1. The measurement setup included an HP ProDesk 400 G6 mini personal computer (PC) [19] running the Windows 11 operating system. A custom-built script, operating with AT commands for communication and data collection, was utilized to interface with the modem, ensuring portable and automated measurements. The PC was configured to automatically power on when connected to the power grid, streamlining the startup process. Additionally, in the event of a power failure, the system was programmed to automatically restart, ensuring continuous operation. A Python program was created for our measurements. It started automatically upon PC startup and conducted comprehensive measurements of all parameters.

The RM502QAEAA-M20-SGASA module from Quectel was utilized for measuring 4G and 5G mobile connectivity. Detailed parameters of the module can be found on the vendor’s website [20]. The module was inserted into the manufacturer’s development board, designated as PCIECARDEVB-KIT [21]. In our measurement configuration, the module was connected to four broadband omnidirectional antennas designed for mobile services, each with a gain of 5 dBi. This configuration enabled support for 4 × 4 Multiple Input Multiple Output (MIMO). To ensure easy portability and protection against damage for both the development board and the RM502QAEAA module, a simple box was created using a 3D printer, as depicted in Figure 1b.

### 3.3. Measured Parameters

Our performance evaluation aimed to characterize and evaluate 4G/5G mobile connectivity in a factory environment comprehensively. To achieve this, we measured all static and dynamic parameters supported by the RM502QAEAA-M20-SGASA module. The static parameters measured, among others, included the RF band on which the operator transmits, the bandwidth used, and the name and location of the transmitter. The dynamic parameters, known as KPIs, are crucial objective measures for evaluating signal quality in mobile networks [12]. Each of these parameters provides information about different aspects of the signal and its reception [22,23]. Below are the definitions and brief descriptions of the considered 4G/5G signal quality indicators:

- **Received Signal Strength Indicator (RSSI)**—RSSI represents the cumulative signal strength received at the device’s antennas, regardless of the signal’s specific sources. Measured in dBm, it considers the overall incoming signal strength, encompassing neighboring base stations’ signals, internal and external interference, and ambient noise. A higher RSSI value indicates stronger signal strength.
- **Reference Signal Received Power (RSRP)**—RSRP quantifies the signal strength received from the present base station in a mobile network. Measured in dBm, its value remains independent of channel width and does not consider spurious signals or interference. A lower RSRP value indicates a weaker signal strength.
- **Reference Signal Received Quality (RSRQ)**—RSRQ, a quantitative parameter introduced by the 3GPP consortium [23], serves as an indicator of the quality of pilot signals received from the current base station. It is calculated using RSSI and RSRP values. A higher RSRQ value (measured in dB) corresponds to superior signal quality.
- **Signal-to-Interference plus Noise Ratio (SINR)**—SINR is calculated as the ratio between the received signal and the combined interference and external noise in the mobile network. It is employed to measure signal quality, denoted in units of dB. A higher SINR value indicates superior signal quality.
- **Channel Quality Indicator (CQI)**—the dimensionless numeric value CQI serves as an indicator of channel quality to the base station. Utilized for adaptive modulation and coding, CQI facilitates the adjustment of transmission rate and efficiency based on signal quality. Reported values of CQI range from 0 to 15, reflecting the level of modulation and coding the UE could employ. Higher CQI values signify favorable conditions in the transmission channel, suggesting the potential for achieving higher transmission rates.

In 4G networks, the utilized module was capable of measuring the following parameters: RSRP, RSRQ, RSSI, SINR, and CQI. However, in 5G networks, the modem only supported the measurement of parameters RSRP, SINR, and RSRQ. The limit values of KPIs indicating the signal quality in an RF channel are provided in Table 1. In this work, we do not distinguish between the limit values for 4G and 5G signal quality [24,25]. Analyzing the correlation between KPIs (i.e., understanding how changes in one KPI may affect others) provides valuable insights into the quality and performance of a cellular network. For instance, higher RSRP values typically correspond to higher RSSI values, indicating a strong overall signal strength. Higher SINR values generally lead to higher CQI values, indicating better channel quality and potential for higher data throughput.

In addition to response time measurements, throughput and latency of data transmission were also measured. Throughput refers to the data transfer rate measured in bits per second (bps) in either uplink (UL) or downlink (DL) communication. Latency, on the other hand, refers to the time it takes for data to travel from the source to the destination and back to the source. It is typically measured in milliseconds (ms). These metrics provide valuable insights into the efficiency and responsiveness of the network.

Response time measurements were conducted on two different commercial servers belonging to major search providers: www.Google.com (accessed on 4 February 2024) and www.Seznam.cz (accessed on 4 February 2024) (search engine in the Czech Republic). The purpose of using these servers was to identify potential issues with the mobile connectivity. Consistently higher response times for one server compared to the other within a specific time frame could indicate a significant load on that particular server. However, these measurements alone do not provide insights into the response time of our mobile network. Consistent response times across both servers indicate the responsiveness of our network, without revealing potential anomalies caused by server overload, whether due to normal traffic or a distributed denial of service (DDoS) attack. Moreover, if one server was available while the other was not, it would suggest a server outage. Conversely, if both servers were down, it would likely indicate a disruption in our mobile connectivity.

### 3.4. Factory-Measurement Environment

The measurement campaigns were conducted in the factory of Tajmac-ZPS, a.s. [27], located in Zlín, Czech Republic. Tajmac-ZPS, a.s. is a leading company engaged in the development, production, and sale of machine tools. The company boasts a comprehensive facility, including a foundry situated directly on the production premises in Zlín, enabling them to cover all stages of machine development and production, starting from mechanical drawing, designing, production of models and castings, their treatment and machining, and up to the final assembly. From the perspective of wireless communication, the factory environment presents challenging conditions for data transmission, characterized by factors such as multipath propagation, noise, and a high volume of moving people [28]. These factors contribute to a rich environment for assessing the performance of wireless networks in real-world industrial settings.

**Figure 2:**
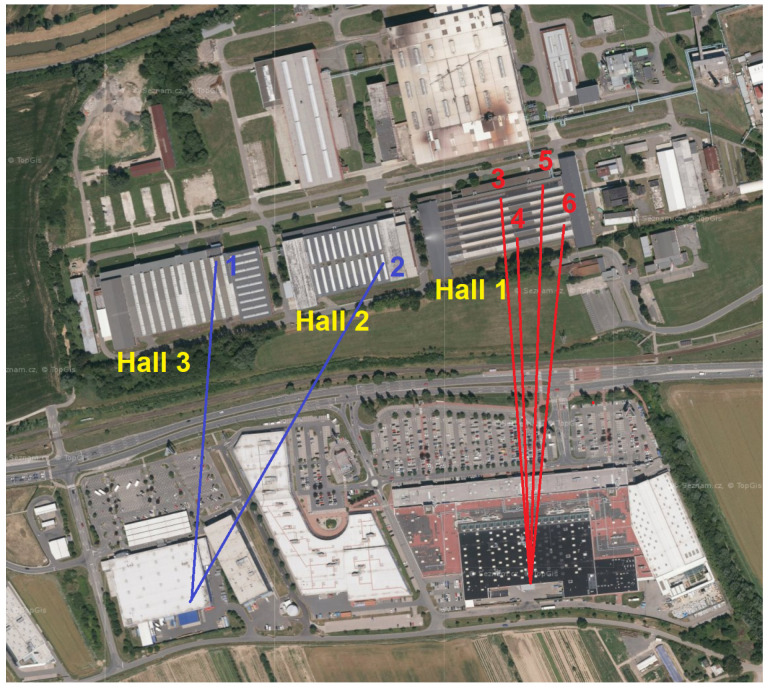
Factory: map view with measurement point and transmitters.

**Figure 3:**
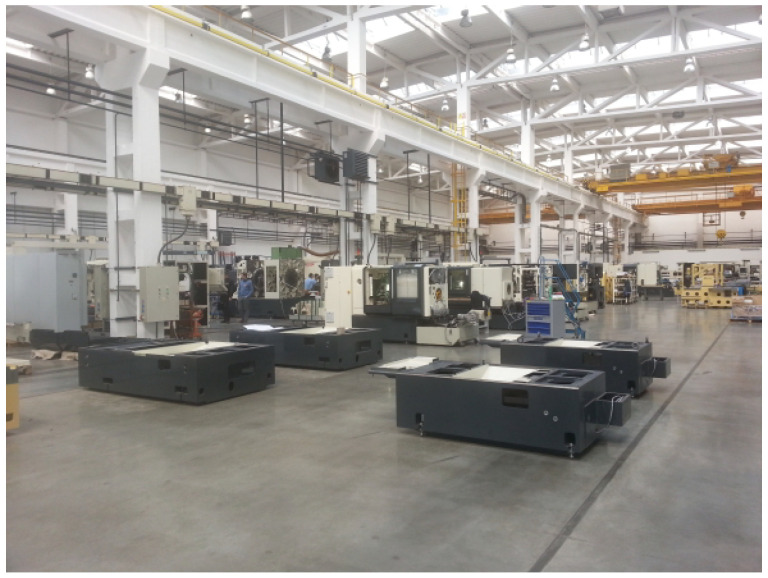
Inside of the factory in which the measurement campaigns were conducted [27].

The measurements were conducted at Tajmac-ZPS, a.s. in three of its halls. Figure 2 illustrates the distribution of individual measuring points and transmitters via an aerial view. The upper half of the picture displays the company premises, while the lower half depicts a large shopping mall where the transmitters, Base Transceiver Station—BTS (Evolved Node B—eNB or Next Generation Node B—gNb) [18,29]—were situated. The distance between the eNB/gNb and our measurement points ranged between 400 and 450 meters. This area was exclusively covered by 5G cellular networks operating at 700 MHz. Based on the current qualitative parameters of the RF signal, the measurement device automatically selected the eNB/gNb to connect to. Throughout the measurements, we were connected to two transmitters, as depicted in Figure 2. Notably, no handovers were observed during the measurement campaign at individual locations. Transitions to different transmitters occurred only with a change of location and a subsequent re-connection to the network. The indoor environment of the factory is depicted in Figure 3. The 4G/5G connectivity measurements were carried out at six measurement locations inside the factory, and a brief description along with an illustrative photo is available in Table 2.

### 3.5. Measurement Procedure

The 4G/5G connectivity measurements were carried out at six measurement locations inside the factory. Locations within the corporate environment where pre-existing Wi-Fi reception issues were encountered were strategically selected. This deliberate choice is central to gaining a deeper understanding of the impact of our 5G infrastructure in regions struggling with Wi-Fi reception issues. The expectation is that locations with Wi-Fi issues are more likely to encounter potential hurdles in receiving 5G signals.

**Figure 4:**
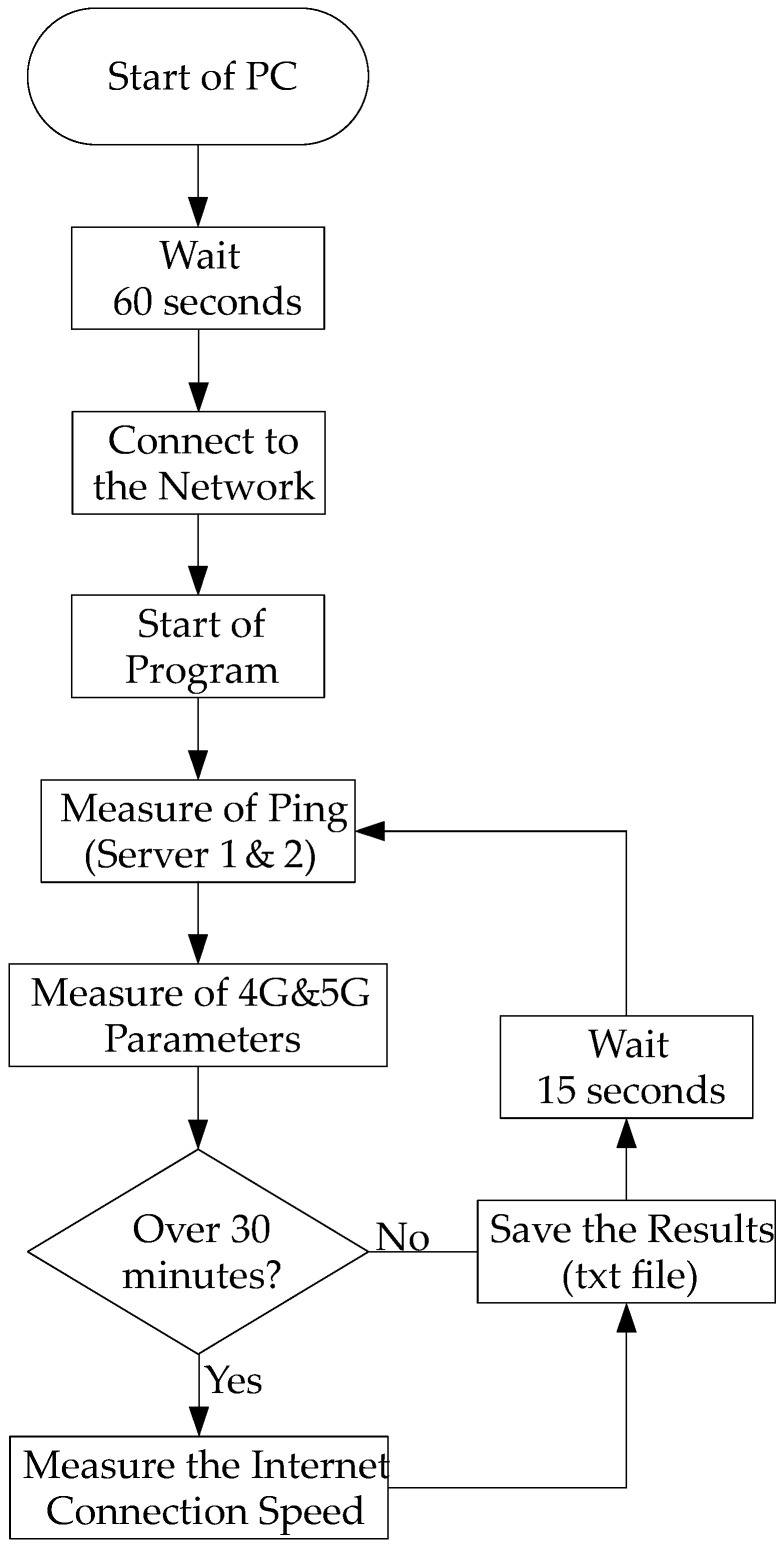
Flowchart of the measurement methodology.

Detailed results from the measurements are presented for a span of 5 days, organized in the following order: Thursday, Friday, Saturday, Sunday, and Monday. This approach enables the observation of the influence of both working and non-working days, and also the impact of so-called weekdays-to-weekends and weekends-to-weekdays on the measurement results. A flowchart of the measurement methodology is shown in Figure 4. The measurements themselves were conducted using AT commands, through which objective parameters described in Section 3.3 were measured. The values of these parameters were recorded every 15 s.

The response of the web server was measured directly from the modem using AT commands. Additionally, the measurement of Internet speed in both directions was performed at half-hourly intervals. However, it is important to note that this measurement frequency is illustrative only, as it was limited due to the Fair User Policy (FUP) data limit applied to the SIM card by the operator (T-Mobile). The infrastructure of the mobile operator T-Mobile was utilized for the measurements, conducted using the well-known server www.speedtest.net (accessed on 4 February 2024). All measurements were conducted from Thursday, 9 March 2023, to Wednesday, 26 April 2023. It is worth mentioning that on Sunday, 26 March 2023, there was a change to daylight saving time (shifting from 2:00 a.m. to 3:00 a.m.), resulting in one hour of missing data in the measurement at location 3. Finally, all the measured and collected data were saved into a txt file for further processing and analysis. The dataset was processed and analyzed offline using MATLAB (online: https://www.mathworks.com/products/matlab.html) (accessed on 4 February 2024).

The dataset we obtained differs from those in [12,13,14] in several aspects. Our dataset primarily comprises measured KPI values, supplemented by details such as cell ID, EARFCN, and uplink and downlink bandwidth related to the physical layer (PHY) of the equipment. In contrast, the datasets in [12,13] include additional information such as residual block error rate (BLER) or medium access control (MAC) throughput, measured on layers other than the PHY. For example, the database in [14] includes information about both lower and higher-layer throughput for 4G. Importantly, these datasets, including ours and those in [12,13,14], were obtained using different measurement equipment. Furthermore, a key distinction lies in the transmission environments in which the datasets were acquired.

## 4. Results

In the case of 4G mobile networks, the measuring equipment was connected to a single cell in LTE band 3 (frequency division duplex, center frequency ≈ 1800 MHz) with a channel bandwidth of 20 MHz. For the 5G NSA network, the measurement equipment was connected to the cell in LTE band n28 (frequency division duplex, center frequency ≈ 700 MHz) with a channel bandwidth of 10 MHz.

As previously mentioned, we conducted measurement campaigns over a span of 5 days at six different locations within the factory. Each dataset for a specific location contains approximately 28,860 samples of KPIs, with 5760 samples collected per day. Consequently, we analyzed around 144,000 samples of KPIs in this work. From this dataset, ≈1% of the data were discounted from processing due to corrupted connections or invalid values.

### 4.1. Statistical Evaluation of the KPIs for 4G and 5G Mobile Networks

**Figure 5:**
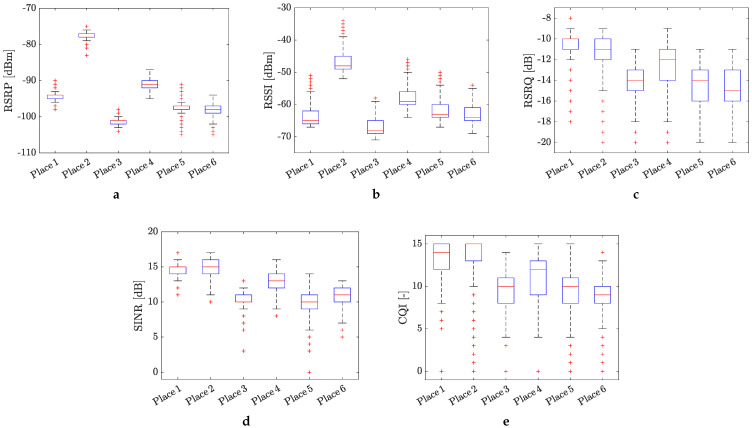
Average values of KPIs for the measured 4G mobile network at different places in the factory: (**a**) RSRP, (**b**) RSSI, (**c**) RSRQ, (**d**) SINR, and (**e**) CQI. Red crosses mark the outlier values.

**Figure 6:**
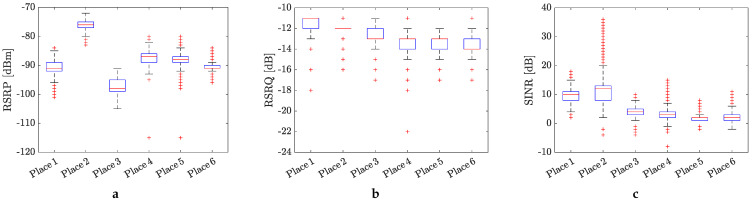
Average values of KPIs for the measured 5G mobile network at different places in the factory: (**a**) RSRP, (**b**) RSRQ, and (**c**) SINR.

The statistical evaluation of the measured KPI values in the form of box plots is presented in Figure 5 and Figure 6, respectively. The obtained results in terms of KPIs clearly shown the influence of the positioning of the measurement equipment on the mobile signal quality. Overall, the RSRP values, repressing the power of the LTE reference signals spread over the full bandwidth and narrowband, indicate moderate 4G mobile signal quality (see Figure 5a). The best (excellent) and worst (bad) signal quality were monitored at places no. 2 and no. 3, respectively. It is interesting that the positioning of the measurement equipment on the top of the electrical panel at place no. 2 practically did not have any negative influence on the values of RSRP.

In contrast to RSRP, the RSSI values (see Figure 5b), representing the cumulative signal strength received at the device’s antennas, indicate almost uniform and good mobile signal quality for all the measured places. Once again, places no. 2 and no. 3 were evaluated as the best and worst, respectively, in the terms of 4G mobile signal coverage.

The statistical evaluation of the RSRQ values, indicating the quality of the received reference signal (as a type of carrier-to-interference ratio), in most cases, shows a 4G mobile signal quality balancing between good and moderate quality metrics (see Figure 5c). Some outlier values can be observed, particularly at the first three locations, indicating poor signal quality. These values were likely measured during occasional minor problems with mobile connectivity, which were mainly monitored at places no. 5 and no. 6.

Figure 5d depicts the statistical distribution of the SINR values, calculated as the ratio between the desired signal and interference. This KPI takes into account various factors that can affect the cellular signal, such as weather conditions, obstacles due to buildings, and incorrect antenna configuration. According to the obtained SINR values, which predominantly result in good and moderate metrics, it appears that these factors do not have a serious influence on the cellular signal quality. All the SINR values are positive, indicating that the 4G network is functioning effectively at the current location.

Lastly, the values of the CQI parameter, serving as an indicator of mobile channel quality, were evaluated (see Figure 5e). According to this parameter, the 4G mobile channel quality at the measured places varied predominantly between good and moderate metrics. However, there is higher observed data dispersion between each sample. The length of the box plot indicates the dispersion of the data: the longer the box, the more dispersed the data. Larger ranges of whiskers indicate a wider distribution, meaning more scattered data. Such scattering of CQI values can be caused by various factors. In our case, it is likely caused by the communication channel being rich in multipath propagation and reflections.

Similar statistical analysis has been provided for the 5G cellular signal quality in terms of RSRP, RSRQ, and SINR, and the results are displayed in Figure 6a–c. Compared to the RSPR values measured for the 4G mobile signal, the RSRP values obtained for the 5G mobile network indicate the stronger power of the LTE reference signals at all places where the measurement were conducted. However, the situation is a bit different for parameters RSRQ and SINR. In both cases, we can observe better cellular signal quality than in the case of 4G mobile signal. It is especially true for SINR, where some negative values indicate that the 5G network may have some short-term outages in the current location. However, the measured 5G mobile signal in both cases is more unified, and in terms of quality, it is comparable for all places where the measurements were conducted.

**Figure 7:**
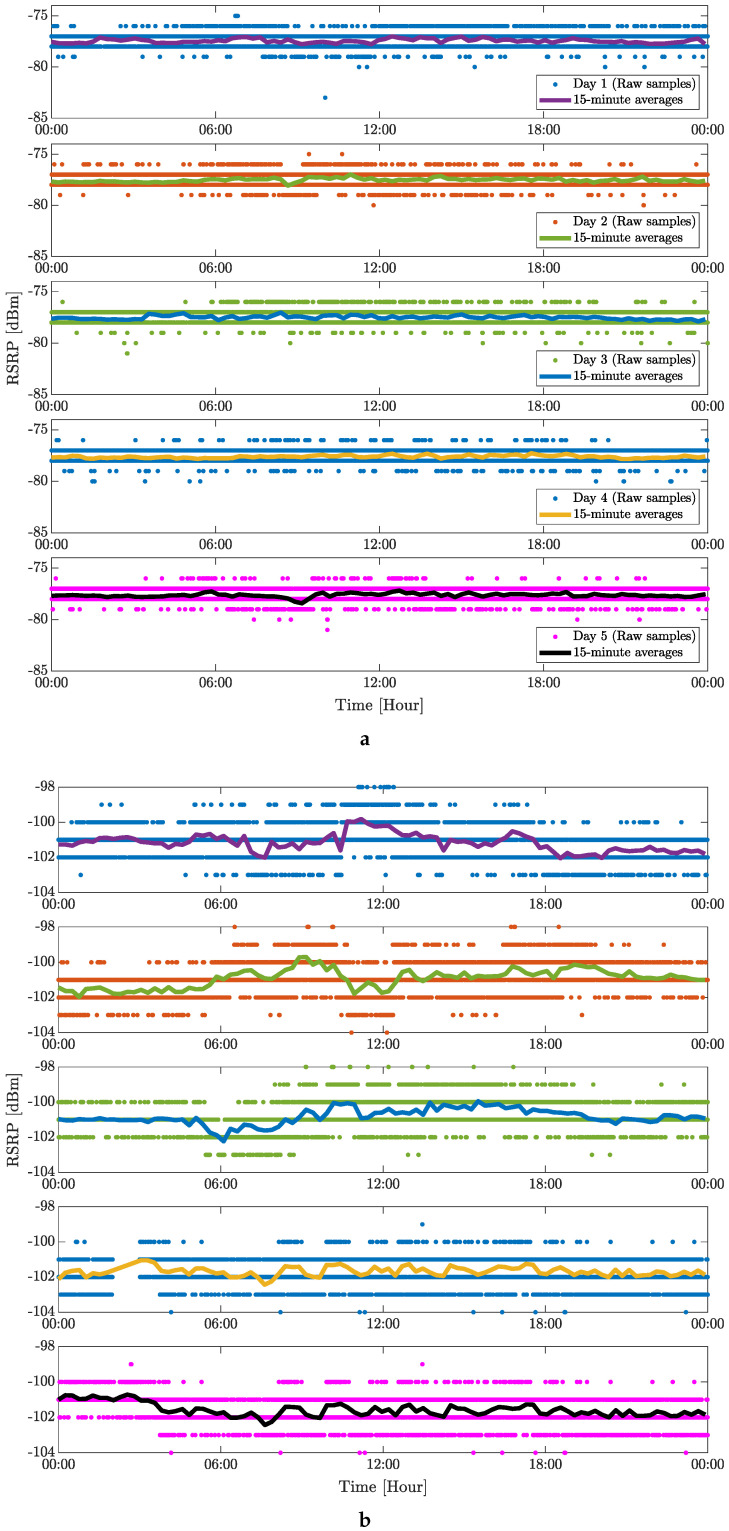
Five days of static indoor (inside of a factory) measurement of 4G mobile signal in terms of RSRP: (**a**) Place no. 2 and (**b**) Place no. 3.

**Figure 8:**
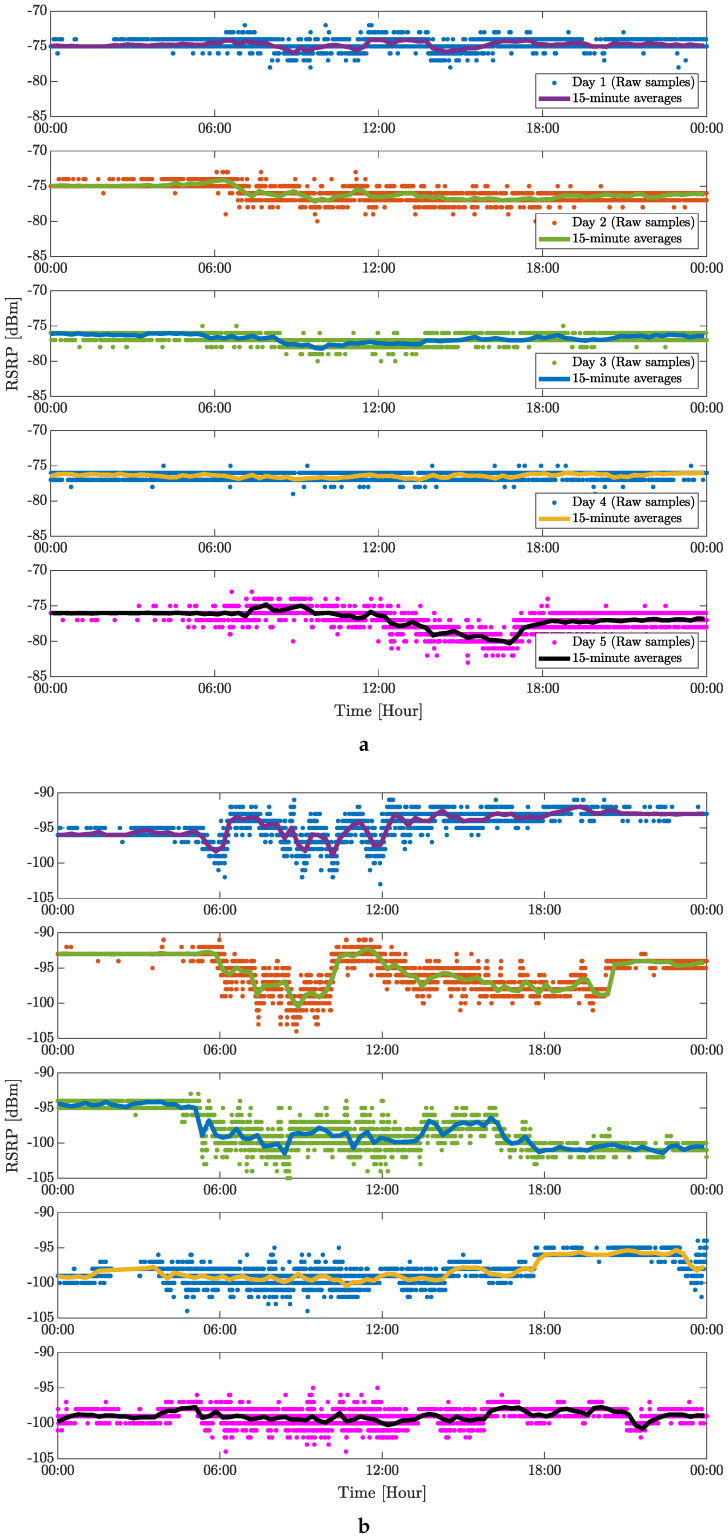
Five days of static indoor (inside of a factory) measurement of 5G mobile signal in terms of RSRP: (**a**) Place no. 2 and (**b**) Place no. 3.

Figure 7 and Figure 8 capture the five-day averages of the measured RSPR values for the 4G and 5G mobile networks, respectively. This representation of the measured data in the time domain is mainly illustrative because the complex evaluation of the measured data is shown in Figure 5 and Figure 6. For place no. 2 (see Figure 7a), RSRP shows almost constant behavior with minima during peak hours (afternoon and evening) and maxima in the off-peak hours (early morning). Long stable intervals are visible, independent of the time of day. More fluctuations in the RSRP values are visible for place no. 3 (see Figure 7b), which can be explained by multipath propagation of the signal, shadowing of the incoming RF signal, or weather conditions [12]. Values measured on the weekend (shown in the last two sub-plots in Figure 7b) show more stable RSRP intervals, predominantly due to almost zero activity in the factory. Similar behavior of the RSRP values is observed for the 5G cellular signal, especially at place no. 2. More visible fluctuations are also observed on the weekend at place no. 3. As assumed in [12], jumps in RSRP values might originate from changes in base station transmitting power. Among other factors (e.g., weather conditions, occupancy of the eNB), Figure 7 and Figure 8 may indicate human movement or structural changes within the facility.

### 4.2. Availability, System Response, and Data Throughput for 4G and 5G Mobile Networks

**Figure 9:**
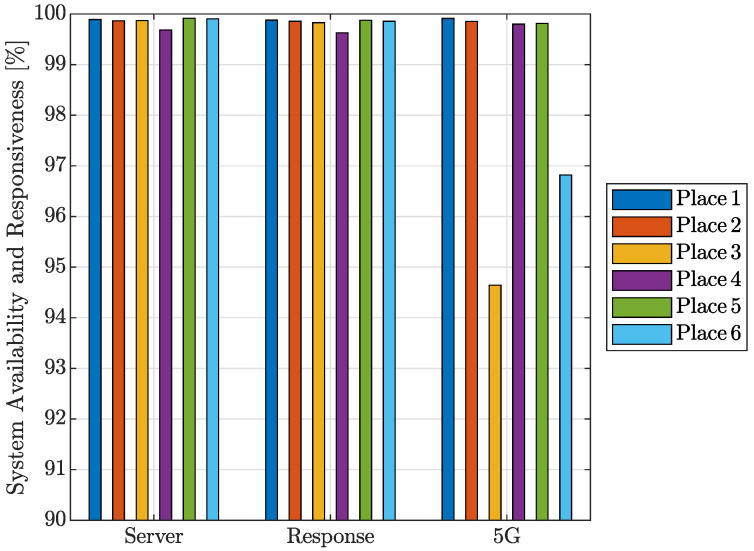
Availability and system response for 4G/5G mobile networks.

The server availability determined from the measurements is shown in Figure 9. The first group of bar graphs, labeled as Server, represents the percentage of times that the server was able to connect when the modem-to-server-to-modem packet travel time was under 2 s. The second group of columns (Response) represents the percentage of time the response was under 100 ms, which can be considered sufficient for any Internet service. Finally, the columns labeled 5G indicate what percentage of the time the measured location had 5G system availability. As can be observed, the 5G system availability was lower than 97% only at places no. 3 and 6.

**Figure 10:**
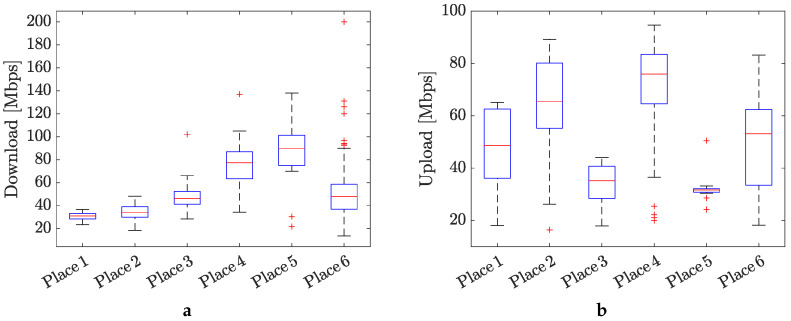
Average speed of the Internet connection measured at different places in the factory: (**a**) download (DL) and (**b**) upload (UL).

Figure 10 shows illustrative Internet connection speeds achieved within the measured places. From the indicative speed tests, we can estimate that the average speed of the Internet connection was around 30–60 Mbps. These speeds fall short of the Internet speeds often associated with 5G systems. The main reason for this discrepancy is the use of a low broadcast frequency with low bandwidth by the commercial operator at the measurement site. Operators cover the Czech Republic extensively with this frequency because it offers the best range due to the lowest signal attenuation over distance from the transmitter.

On the other hand, the speeds achieved are sufficient for a full machine connection to the 5G network and sending large amounts of data.

**Figure 11:**
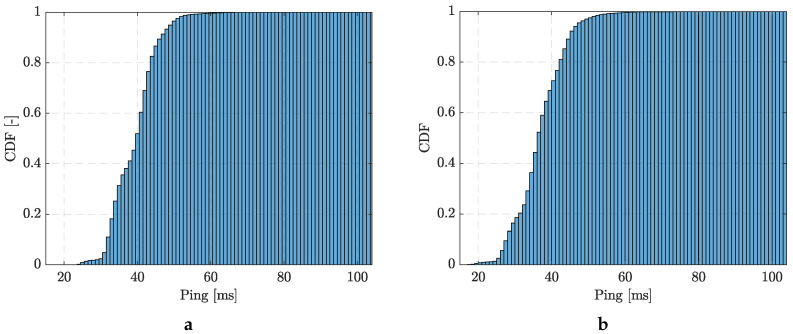
Server response distribution function for (**a**) Google server and (**b**) Seznam server.

The Cumulative Distribution Function (CDF) [30] of server responses measured at two different places is shown in Figure 11. From the perspective of system response, i.e., the time it takes for the measurement packet to travel from the device to the server and back, it can be observed that there were no fluctuations. The response time averaged approximately 40 ms, a low enough value for universal use. This response time was consistent across different times and locations, regardless of the associated signal strength.

The obtained results confirm that indoor environments, especially a heavy industry environment, pose unique challenges to signal coverage, including signal penetration through building materials, multipath propagation, and interference from electronic devices. The conducted experiments have revealed that the placement of the measurement equipment (refer to Table 2) also influences the values of KPIs. Based on the measurement results, place no. 3 poses a challenge to signal coverage due to the characteristics of the indoor environment (refer to the description in Table 2). From this point of view, other places should not be critical.

## 5. Conclusions

This paper focused on on analyzing the 4G and 5G cellular signal coverage, through long-term measurement campaigns conducted in a complex factory environment. To achieve this goal, we proposed and implemented a simple and portable measurement setup along with an appropriate measurement methodology.

During the measurements, no significant events were observed that significantly affected the radio data transmission over the 5G network. This suggests that the 5G system is quite robust and resistant to interference from industrial operations within the factory. With the exception of Place no. 2, where signal strength was weak, the system remained connected to the 5G network at all times. In cases where 5G connectivity failed, the system seamlessly switched to the 4G network, ensuring continuous connectivity without any complete loss of service. During the measurements, the system consistently operated within the n28 band, corresponding to a frequency of 700 MHz, without any changes in frequency band observed. Additionally, there were no instances of the system switching from one transmitter to another during the measurements at individual sites. The only occurrence of switching transmitters was when the device was moved to a different measurement site and powered back on.

The observed changes in signal strength and quality between night and day are likely due to variations in the number of devices connected to the transmitter, rather than changes in traffic or interference within the industrial plant. These fluctuations are noticeable even during weekends, indicating that they are not solely related to weekday activity. The transmitters, located in large shopping malls, experience high user connectivity during weekdays and weekends but significantly fewer users at night. Additionally, there was a notable difference of approximately 20 dB in signal strength between the location with the highest and lowest signal strengths, despite both locations being similarly distant from the transmitter. This suggests that the receiver’s location within the building significantly influences the received signal strength. There are several strategies to avoid low signals and fluctuations in a complex factory environment, including the following: signal monitoring (prompt detection of low signals or fluctuations, enabling proactive measures to address them before they impact operations), signal amplification (use of signal amplifiers or boosters to strengthen weak signals in areas where coverage is poor), and antenna orientation (properly orienting antennas can improve signal reception).

In the future, our focus will be on conducting long-term benchmark tests to gather extensive 4G/5G-based data from both indoor and outdoor environments. We plan to develop an application dedicated to efficiently collecting and processing data [31]. Additionally, we aim to implement algorithms that will enhance the forecasting capabilities of 4G/5G mobile signal coverage, enabling better prediction of signal quality and coverage in various scenarios [32,33,34].

## Figures and Tables

**Table 1:** Limit values of the KPIs indicating the conditions in the RF channel [24,26].

Metric	Excellent	Good	Moderate	Bad
**RSSI**	>−65 dBm	−65 to −75 dBm	−75 to −85 dBm	<−85 dBm
**RSRP**	>−80 dBm	−80 to −90 dBm	−90 to −100 dBm	<−100 dBm
**RSRQ**	>−10 dB	−10 to −15 dB	−15 to −20 dB	<−20 dB
**SINR**	>20 dB	13 to 20 dB	0 to 13 dB	<0 dB
**CQI**	13 to 15	10 to 13	6 to 10	1 to 6

**Table 2:** Description of the measurement places. The red arrows indicate the location of the measurement equipment.

Place and Date of Measurement	Photo	Description
**Place 1** (9–13 March 2023)	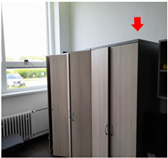	The measurement was conducted in the office located in hall number 3. The measurement equipment was positioned on the opposite side of the hall from the transmitter. This hall serves as a transshipment center for products.
**Place 2** (15–19 March 2023)	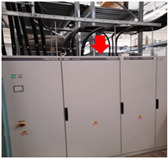	The measurement was conducted in the office located in hall number 2. The measurement equipment was positioned on top of the electrical panel.
**Place 3** (22–26 March 2023)	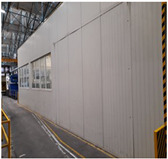	The measurement was conducted inside the metal cell in hall 1, which houses the company’s production facilities.
**Place 4** (29 March–3 April 2023)	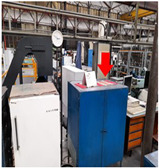	The measurement was conducted in hall number 1, within the metal cabinet situated between the production areas.
**Place 5** (8–12 April 2023)	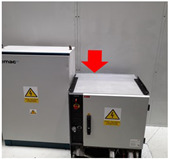	The measurement was conducted in hall number 1, within the sheet metal cabinet located at the production cell.
**Place 6** (22–26 April 2023)	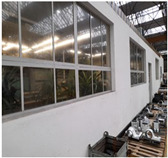	The measurement was conducted in hall number 1, inside the brick shed located within the hall. The measurement equipment was placed on a shelf at a height of 1 meter.

## Data Availability

The data presented in this study are openly available on GitHub: https://github.com/polak-l/4G-5G-Mobile-Signal-Coverage-in-a-Factory (accessed on 4 February 2024).
